# Vacancy Defects and Multilayer Shading in Graphene Monolayers: Enhancing Proton Transport in Centimeter-Sized Direct Methanol Fuel Cells

**DOI:** 10.1021/acsami.5c16491

**Published:** 2025-11-05

**Authors:** Weizhe Zhang, Xiaoting Liu, Buhang Chen, Luzhao Sun, Zhongfan Liu, Grégory F. Schneider

**Affiliations:** † Leiden Institute of Chemistry, Faculty of Science, 4496 Leiden University, Einsteinweg 55, Leiden 2333CC, Netherlands; ‡ Center for Nanochemistry, Beijing Science and Engineering Center for Nanocarbons, Beijing National Laboratory for Molecular Sciences, College of Chemistry and Molecular Engineering, Academy for Advanced Interdisciplinary Studies, 12465 Peking University, Beijing 100871, China; § Beijing Graphene Institute (BGI), Beijing 100095, China

**Keywords:** graphene, proton exchange membrane, fuel cell, graphene defects

## Abstract

Proton selectivity of monolayer graphene offers promising opportunities in energy devices. The impermeability of graphene to methanol, on the other hand, offers potential as a membrane in direct methanol fuel cells (DMFCs). The integration of graphene in DMFCs requires samples larger than 1 cm^2^ and therefore must be grown via chemical vapor deposition (CVD) instead of using exfoliation. CVD graphene—as opposed to exfoliated flakes—however, contains inherent defects and multilayer patches, both of which could be harnessed to tune the fuel cell performance controllably. Here, we investigated the impact of multilayer patches on the performance of centimeter-scale graphene films in DMFCs. While single-crystalline graphene (SCG) has no multilayer patches, polycrystalline graphene (PCG) can exhibit an areal ratio of multilayer patches up to ∼4%. While multilayer patches are less reactive to plasma etching, the monolayers within SCG and PCG exhibit similar reactivity, enabling precise control over the etching process. These shaded areas of the membrane contribute to higher proton selectivity, likely due to more constrained and controlled defect sites. Our findings indicate that plasma-induced defects yield a proton conductance 10% higher than pristine graphene, and we attribute the enhanced performance of defected PCG to the shading effects provided by the multilayer patches.

## Graphical abstract

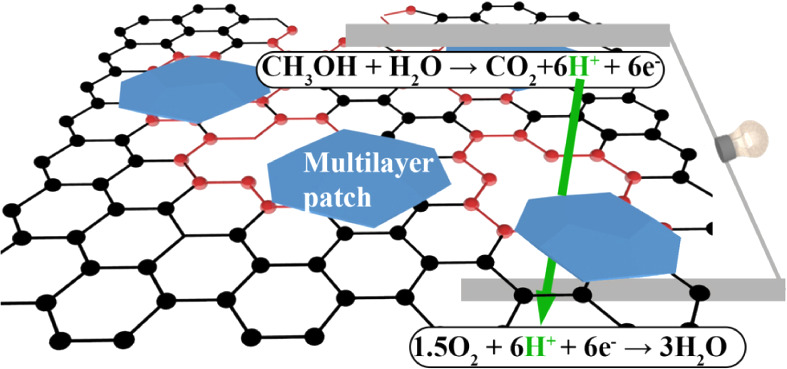

## Introduction

Monolayer graphene, obtained through mechanical exfoliation and known for its near-perfect 2D crystal structure, has demonstrated an areal conductance of approximately 3 mS cm^–2^ at room temperature when measuring ionic current within proton-rich proton exchange membranes^1,2^ . However, in fuel cell applications, the conductance of the membrane must exceed 1 S cm^–2^ to compensate for the significant resistive loss in the circuit and maintain a sustainable power output.^3^ Moreover, the micrometer size of the exfoliated graphene flake complicates upscaling to practical devices. Chemical vapor deposition (CVD) grown graphene therefore provides a centimeter-scale source of monolayer graphene with the possibility for subsequent functionalization.

The remarkable proton selectivity of graphene, through size-sieving channels, has the potential to reduce the limitations faced in direct methanol fuel cells (DMFCs), particularly because polymeric membranes require efficient charge carrier selectivity. DMFCs, being liquid fuel cells, present a promising alternative to hydrogen fuel cells, especially due to the advantages offered by methanol as a fuel source.^4^ However, the trade-off between selectivity and conductivity for the conventional polymer proton exchange membranes is a significant limitation for the practical application of liquid fuel cells.^5,6^ Unlike ions with charge, methanol, as a neutral molecule with a diameter of only 0.36 nm, represents one of the major challenges for promoting proton transport and preventing fuel crossover in polymeric membranes, specifically, for cation-exchange membranes,^7^ without sacrificing conductance.^8,9^ In a DMFC, the membrane serves as an insulating barrier between the electrodes and selectively allows the passage of charge carriers (protons in acidic fuel cells) while preventing fuel crossover from the anode to the cathode.^10^ In fact, upon crossover, methanol would oxidize at the cathode, competing with oxygen reduction, therefore leading to a decrease in cell voltage and generating carbon monoxide that poisons the catalysts.^9^ Various strategies, such as incorporating additional layers and components, have been employed to improve proton/methanol selectivity, but they often result in reduced proton conductance.^11,12^

CVD graphene sandwiched between two Nafion supports has shown to have a proton conductance upto a thousand times higher (29 S cm^–2^) compared to exfoliated graphene. Bukola et al. reported a net proton conductance of 29 S cm^–2^ through CVD graphene, sandwiched between two Nafion layers.^13^ Polycrystalline graphene and graphene with defects and grain boundaries can exhibit proton conductance comparable to Nafion, although the selectivity for protons vs neutral molecules and cations is usually less pronounced due to the prevalence of defects.^14^ −^15,16,17^ Faster proton transport through graphene can occur at grain boundaries and defects, albeit with different conductance and selectivity.^3,14,18–22^ Separately, CVD graphene was also used as an additive layer in bulk Nafion polymeric membranes and was tested in DMFCs:^23,24^ the addition of a graphene layer to the polymer membrane efficiently suppresses the methanol crossover permeation. However, the formation of defects and cracks is inevitable during the operation of embedding the graphene single layer with a swelling Nafion layer or within the membrane electrode assembly (MEA) process where rough catalyst surfaces are integrated. This brings the question of whether graphene could be a standalone membrane,^17^ leveraging its unique properties of crystallinity, and whether controlling the defect types and density would influence proton transport and fuel cell performance. Besides the subsequent distortion introduced during the MEA, the “quality” of CVD graphene depends on growth conditions, including domain size, the presence of pinholes, and the amount of multilayers, compared to mechanically exfoliated graphene. Therefore, CVD graphene represents an ideal platform to understand the correlation between the inherent property of defects at the level of centimeter-sized membranes and the performance as a proton conductive membrane in, for example, a DMFC as studied here.

In this work, we investigated the influence of multilayer patches in polycrystalline vs multilayer-free single-crystalline CVD graphene and the influence of subsequent nitrogen doping on proton transport in DMFCs. By deliberately introducing defects through plasma exposure, we observed that the presence of multilayer patches improves the temperature tolerance with respect to the methanol crossover. Notably, after the introduction of defects, the graphene with multilayer patches achieved a similar proton conductance compared to multilayer-free graphene. Multilayer areas act as shading patches for defects introduction, but—importantly—play a crucial role in mechanically stabilizing the graphene monolayer during assembly within the MEA process. This shading effect highlights the importance of the relationship between the proton permeability of graphene and the membrane performance, at least in DMFCs, and opens new possibilities to improve the stability of CVD graphene membranes, by changing CVD growth procedures.

## Results and Discussion

### Single-Crystalline Graphene vs Polycrystalline Graphene as Proton-Selective Membranes

The growth of graphene through chemical vapor deposition (CVD) allows for control over its crystallinity,^25^ which in turn affects the presence of inherent defects and multilayers. Figure 1A presents optical images and Raman characterizations of graphene grown as a single-crystal (SCG) and commercialized polycrystalline graphene (PCG) on a SiO_2_/Si substrate. The Raman spectrum reveals the G band at approximately 1580 cm^–1^ and the 2D band at around 2700 cm^–1^, which provides information about the layer numbers and integrity of graphene sheets. Areas with an intensity ratio of I_2D_/I_G_ close to 2 indicate monolayer regions, while other areas refer to multilayers.^26–28^ In the optical images, multilayer regions are distinguishable as island-like patches in PCGs, confirmed by a Raman spectrum with I_2D_/I_G_ ∼ 1. In contrast, SCG exhibits minimal presence of such island-like patches, indicating a predominance of multilayer-free regions. The presence of the D peak (∼1360 cm^–1^), associated with lattice disorder in graphene, is observed in PCGs, further indicating the existence of inherent defects.^26,28^ To quantify the ratio of multilayer patches, we conducted a statistical analysis of their coverage area, determining the areal ratio as the percentage of the total surface area occupied by multilayer patches in PCGs (Figure 1B). The average ratio is approximately 4.1 ± 1%. Figure 1C demonstrates the homogeneity of SCG and PCG through I_2D_/I_G_ Raman mapping, supporting the identification of distributed patches as island-like multilayers.

**Figure 1:**
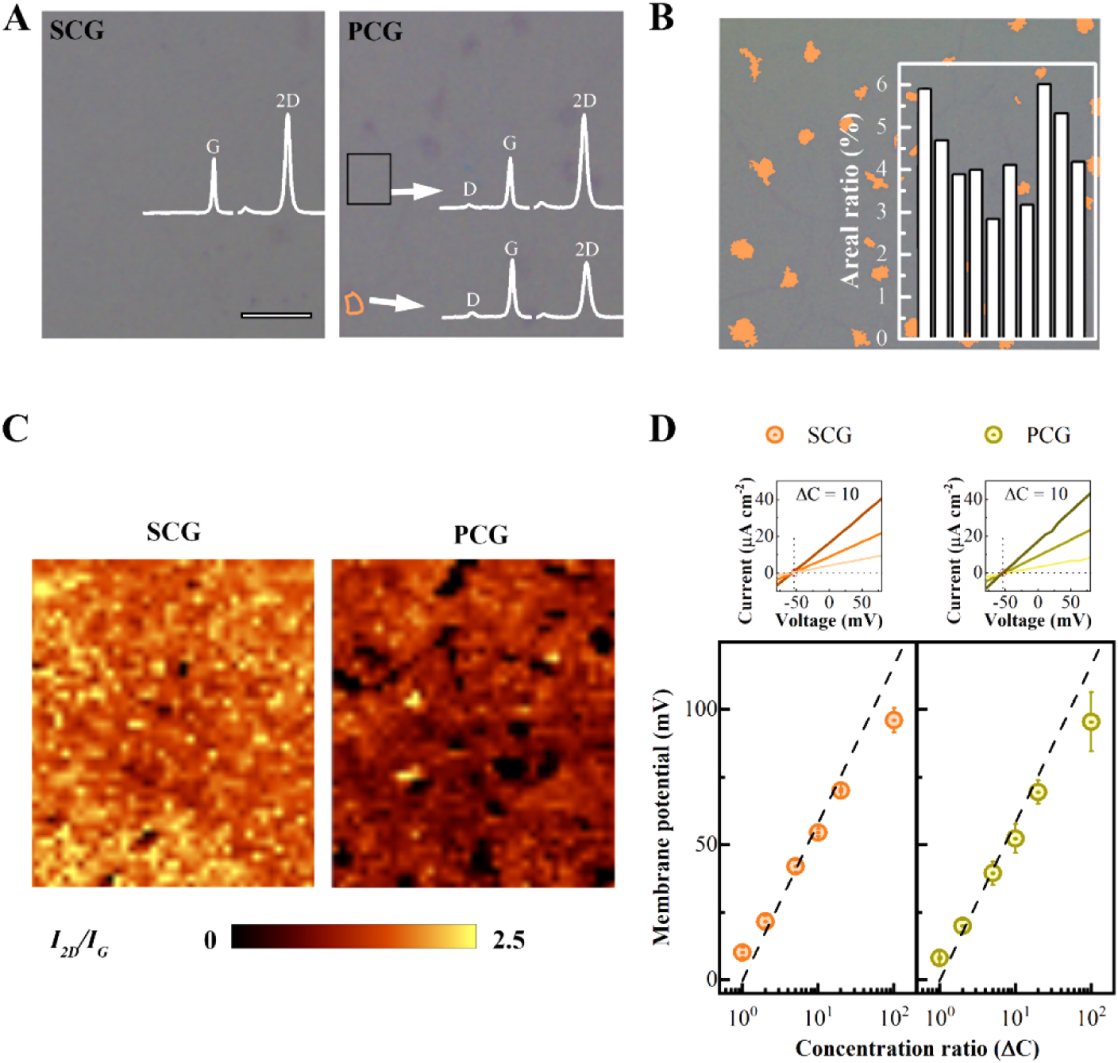
Raman characterization, multilayer analysis, and cation/anion selectivity of single-crystalline graphene (SCG) and polycrystalline graphene (PCG). (A) Optical images of SCG and PCG on a SiO_2_/Si substrate. Insets: Raman spectra of different regions. Unlike SCG, which exhibits consistent spectra over the entire area, PCG shows the presence of multilayer domains alongside the monolayer regions, with more noticeable D peaks. Scale bar: 5 μm. (B) Analysis of patch-like multilayer domains in PCG, marked to determine their areal ratio, which is used to quantify the area covered by multilayer patches. Inset: Areal ratio of randomly selected areas across ten independent samples. (C) Intensity ratio, I_2D_/I_G_, of the same area as in (A), indicating the distribution of multilayers and the homogeneity over the area. (D) Measurement of membrane potential in a flow cell setup, with the SCG and PCG membranes placed between two reservoirs filled with HCl solutions of varying concentration ratios. The selectivity toward chloride ions was estimated using the Nernst equation, and the theoretical maximum membrane potentials of a proton-selective membrane are shown as a dashed line. Top: current (I)–voltage (V) curves of membranes with multilayer-free graphene and graphene with multilayers under a concentration ratio of ΔC = 10, with reservoirs filled with HCl concentrations of 1–0.1 M, 0.1–0.01 M, and 0.01–0.001 M.

To evaluate graphene as a proton-conductive membrane, we transferred graphene sheets onto a polycarbonate porous filter membrane. The topside was spin-coated with a Nafion layer to serve as both a stabilizer and an electrical insulator, and the membrane assembly was sealed with PTFE gaskets (Figures S1–S3). The rigid polycarbonate support helps mitigate swelling effects on graphene, in contrast to graphene directly sandwiched between two Nafion layers.^29^ To verify membrane integrity, we conducted tests on proton selectivity toward chloride by placing the membrane between two chambers filled with HCl at different concentrations. By creating a concentration difference, we could measure the membrane potential at zero current and compare it with the theoretical potential assuming perfect proton selectivity. The theoretical potentials were calculated using the Nernst equation:

1 $$V_{0}=\left(t_{\mathrm{Cl}}-t_{H}\right)\left(k_{B}T/e\right)\ln\left(\Delta C\right)=-\left(2t_{H}-1\right)\left(k_{B}T/e\right)\ln\left(\Delta C\right)$$

where t_Cl_ and t_H_ are transfer numbers of Cl^–^ and H^+^, k_B_ is the Boltzmann constant, T is the temperature, e is the elementary charge and ΔC is the concentration ratio. Figure 1D shows membrane potentials measured with membranes of SCGs and PCGs with the dashed lines indicating the theoretical membrane potential calculated for the perfect H^+^/Cl^–^ selectivity based on eq 1. In the absence of graphene, no selectivity was observed, indicating that the Nafion layers used in the study did not provide the expected proton selectivity for the thin spin-coated Nafion layer. The membrane potential with a membrane exhibiting perfect proton selectivity toward chloride was measured at 58 mV for a concentration ratio of ΔC = 10. In the absence of graphene, no membrane potential was observed, indicating that the thin porous Nafion ionomer layers used in the study allowed unrestricted electrolyte permeation. When graphene was introduced, we observed bias potentials which were dependent on concentration ratios. With the balanced concentration of 0.1 M of HCl on both sides of the graphene membrane, we observed a bias potential of 5–10 mV. This is likely because of the asymmetric structure and charge inhomogeneities from the Nafion on both sides of the membrane. The membrane potential for PCG is approximately 48 mV, similar to SCG, which is around 47 mV, suggesting that graphene plays the dominant role in establishing membrane selectivity by impeding free electrolyte exchange. In more detail, the negatively charged sulfonate groups in the Nafion ionomer may contribute to selective transport by repelling anions such as Cl^–^, thereby enhancing proton selectivity. However, given that both Nafion and graphene exhibit high selectivity for protons over chloride ions, the observed membrane potentials, lower than the ideal value of 58 mV for a perfectly proton-selective membrane, imply some degree of chloride permeation. This lower membrane potential likely arises from defects in the graphene, which compromise its ion-selective barrier properties. This lower membrane potential compared to a perfectly proton-selective membrane suggests the occurrence of chloride permeation through the defects in graphene. Subsequently, the as-prepared graphene composite membranes were tested in a DMFC, with Pt/Pd@C alloy as the anode catalyst and Pt@C as the cathode catalyst.

### DMFC Performance with Pristine Graphene

We measured DMFCs with three independent samples, each set with SCG and PCG respectively to understand how the multilayer patches influence the proton conductance and membrane stability. Figure 2A presents the cell voltage (V) to current density (I) curves (V–I) and power density (P) to current density curves (P–I) of SCG (sample 1) and PCG (sample 1) at 60 °C. The maximum power output of SCG is 45.7 mW cm^–2^, approximately double compared to PCG (21.4 mW cm^–2^) at the same temperature (60 °C). Two additional samples were studied, as shown in Figure 2B. SCG-DMFCs exhibited an average maximum power density of 18.2 ± 4.4 mW cm^–2^, while PCG-DMFCs yielded an average maximum power density of 11.3 ± 0.67 mW cm^–2^ at room temperature. At a higher temperature of 70 °C, this difference was reduced to 15% (34.8 ± 18.6 mW cm^–2^ for SCG and 30.0 ± 12.3 mW cm^–2^ for PCG). In contrast to the observable decrease in maximum power output for SCGs (sample 2 and 3) above 50 °C, the maximum power output of graphene with multilayers gradually increased with a lower standard deviation among samples. Since the maximum power output is a parameter where both proton conductance and methanol crossover are intertwined, we also separately estimated them both.

**Figure 2:**
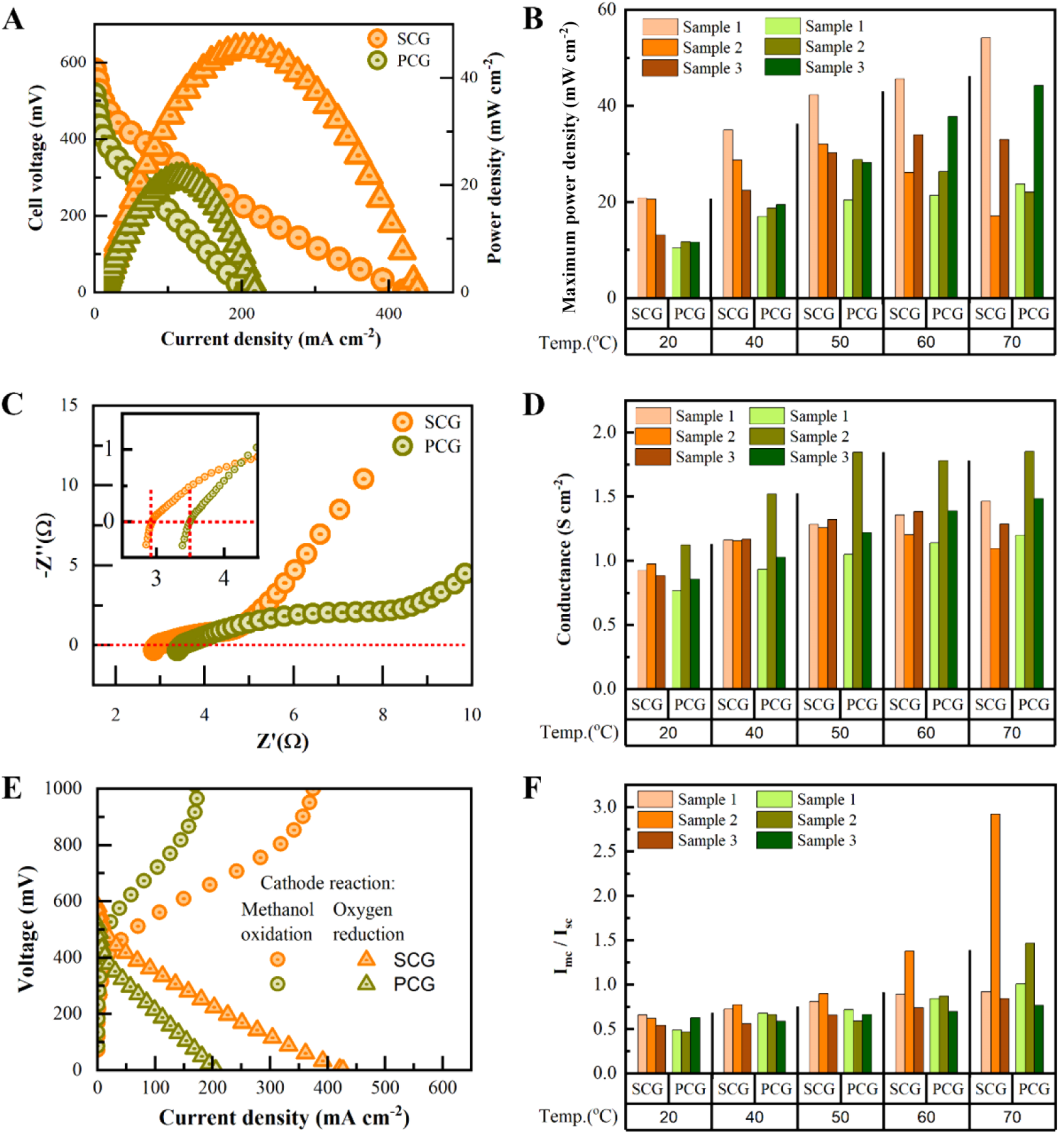
DMFC performance of pristine SCG and PCG. (A) Cell voltage (V) to current density (I) curves (V–I) in circle symbols, and power density (P) to current density curves (P–I) in triangle symbols for SCG-DMFC and PCG-DMFC at 60 °C, using a 1 M methanol/water solution. (B) Maximum power density of three samples of SCG and PCG at various operating temperatures ranging from 20 to 60 °C. (C) Nyquist plots obtained from electrochemical impedance spectroscopy (EIS) of SCG and PCG at open-circuit voltage at 60 °C. The intercepts on the *X*-axis represent the membrane resistance. Inset: zoom-in of the low-frequency area for better visibility of the intercepts. (D) Areal conductance of SCGs and PCGs. (E) Linear sweep voltammetry curves of SCG-DMFC and PCG-DMFC for methanol oxidation and cell output. The maximum methanol crossover rate is indicated by the peak methanol oxidation current (I_MC_) permeating from the anode. The short-circuit current (I_SC_) is used to analyze the correlated effects of methanol crossover and proton conductance. (F) I_MC_/I_SC_ ratio of SCGs and PCGs.

To do so, the membrane conductance was characterized using electrochemical impedance spectroscopy (EIS). Figure 2C displays the Nyquist plots of SCG (sample 1) and PCG (sample 1). The intercept at the *X*-axis of the Nyquist plots represents the membrane resistance, with the electrolyte resistance being negligible in the MEA system. For ease of comparison, the membrane resistances were converted into areal conductance by applying Ohm’s law (Figure 2D). The averaged membrane conductance of SCG was 0.93 ± 0.05 S cm^–2^, while the averaged membrane conductance of PCG was 0.85 ± 0.11 S cm^–2^ at room temperature, indicating lower proton conductance for PCG. The power outputs of both SCG and PCG indicate a much higher proton conductance than exfoliated graphene (3 mS cm^–2^). This suggests that proton transport through CVD graphene primarily occurs via defects. However, the conductance of SCGs (1.28 ± 0.18 S cm^–2^) was approximately 85% that of PCG (1.51 ± 0.33 S cm^–2^) at 70 °C. Since the samples were freshly prepared and measured under the same conditions, we assumed that the variations in proton conductance correspond to changes in the methanol crossover rate, which influences the catalytic processes.

To therefore estimate the methanol crossover, methanol molecules that permeated from the anode to the cathode were oxidized by applying a voltage (Figure 2E and Methods section), while oxygen was purged away using a constant nitrogen flow. The peak of the methanol oxidation current corresponds to the maximum methanol crossover rate (I_MC_). The ratio of I_MC_ to the short-circuit current (I_SC_), I_MC_/I_SC_, indicates the rate of methanol crossover relative to proton conductance, which illustrates the combined effects of methanol crossover permeation and proton transport. Figure 2F shows the I_MC_/I_SC_ ratios of all samples at operating temperatures ranging from 20 to 70 °C. Remarkably, the I_MC_/I_SC_ ratio of SCG sample 2 increased dramatically from 50 to 70 °C (from 0.90 to 2.92), indicating more severe methanol permeation at higher temperatures for this sample. This explains the decrease in power output and conductance observed for this sample at 70 °C. On average, the I_MC_/I_SC_ ratio of SCGs (1.56 ± 1.18) was approximately 44% higher than that of PCGs (1.08 ± 0.36) at 70 °C.

In summary, it was observed that SCGs exhibited higher power outputs compared to PCGs, attributed to their higher proton conductance. This enhanced conductance may stem from defects introduced during membrane fabrication and operation, as we observed significant variation among individual samples. Although inherent defects were observed in PCGs through Raman spectroscopy, their lower proton conductance suggests that multilayer patches likely hinder proton transport. Furthermore, the conductance of PCGs showed a larger increase with increasing operating temperature compared to SCGs. Moreover, the increase in temperature led to a significant increase in methanol permeation for one of the SCGs, while the observed increases in methanol permeation for PCGs were more gradual with the temperature. This suggests that PCGs exhibit greater stability at higher temperatures (50 to 70 °C). These findings imply that SCGs are more susceptible to defect-related changes under operational conditions, while PCGs maintain more consistent properties. To further investigate the role of defects, the following section focuses on membrane performance using samples with intentionally introduced defects, allowing for a comparison of the intrinsic differences between SCGs and PCGs.

### Defected Graphene and DMFC Performance

Pristine graphene membranes are poorly proton conductive. There is thus a need for strategies to enhance proton conductance and explore how the multilayer patches affect proton transport and methanol crossover. Introducing defects deliberately by plasma exposure is a common strategy to introduce defects into graphene.^16,21^ We used a nitrogen plasma etching procedure to create defects in graphene.^30^

Figure 3A depicts nitrogen-doped single-crystalline graphene (ND-SCG) after nitrogen plasma exposure. The Raman spectrum reveals a decrease in the intensity ratio of the 2D peak to the G peak (I_2D_/I_G_) to approximately 0.43 (Figure 3B), indicating the presence of defects. Moreover, the emergence of a prominent D peak with an intensity ratio of the D peak to the G peak (I_D_/I_G_) of around 2 (Figure 3C) further confirms the introduction of defects in ND-SCG. Similarly, nitrogen-doped polycrystalline graphene (ND-PCG, Figure 3D) also exhibits a reduced I_2D_/I_G_ ratio of approximately 0.4 (Figure 3E) and an increased I_D_/I_G_ ratio of about 2 (Figure 3F). It is worth noting that the defect densities in SCG and PCG are found to be very similar, enabling us to compare the performance of DMFCs solely based on the presence vs absence of multilayer patches.

**Figure 3:**
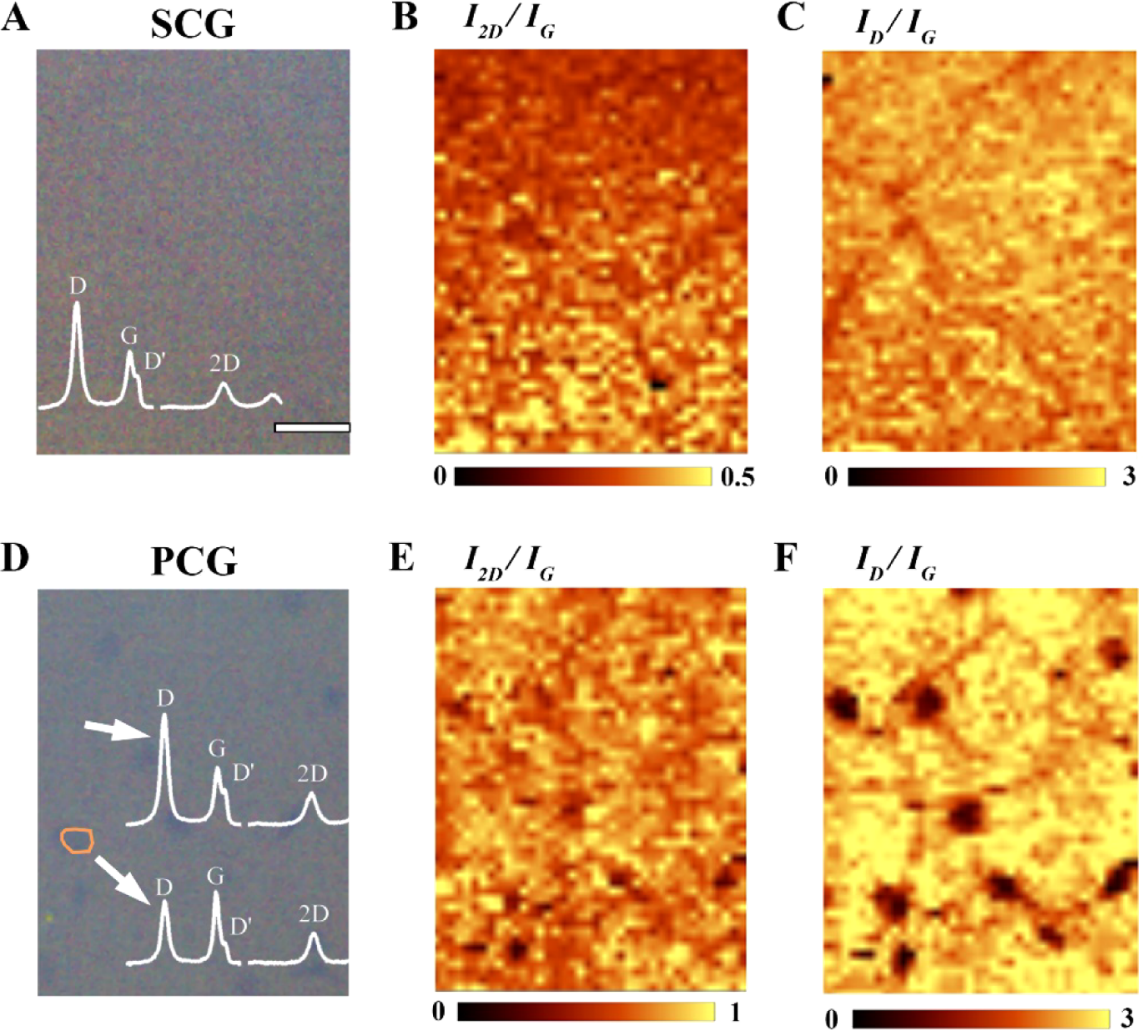
Plasma-treated SCG and PCG. (A) Optical image of SCG on a SiO_2_/Si substrate after nitrogen plasma exposure. The scale bar is 5 μm. (B, C) Raman mapping of I_2D_ /I_G_ and I_D_ /I_G_ of the same area as in (A). (D) Optical image of SCG on a SiO_2_/Si substrate after nitrogen plasma exposure. The scale bar is 5 μm, the same as in (A). (E, F) Raman mapping of I_2D_ /I_G_ and I_D_ /I_G_ of the same area as in (D). Notably, the color bars in (B, E) are different for better contrast.

Figure 4A presents the conductance of three ND-SCG samples at operation temperatures ranging from 20 to 70 °C, with the dashed line indicating the baseline conductance of pristine SCGs at the specific operation temperature. The higher conductance of ND-SCGs, compared to SCGs, indicates the effect of defects on the observed increase of the proton conductance. Specifically, at 50 °C, the average conductance of ND-SCGs is 1.76 ± 0.11 S cm^–2^, which is 36% higher than that of SCGs. Across the entire range of operating temperatures, the conductance of ND-SCGs surpasses that of SCGs by 36–54%. Accordingly, the increased maximum power output was also observed with ND-SCG (Figure 4B). At 50 °C, the average maximum power output of ND-SCGs is 40.0 ± 8.2 mW cm^–2^, representing a 15% increase compared to SCGs. At 70 °C, the maximum power output of ND-SCGs surpasses that of SCGs by 21%.

**Figure 4:**
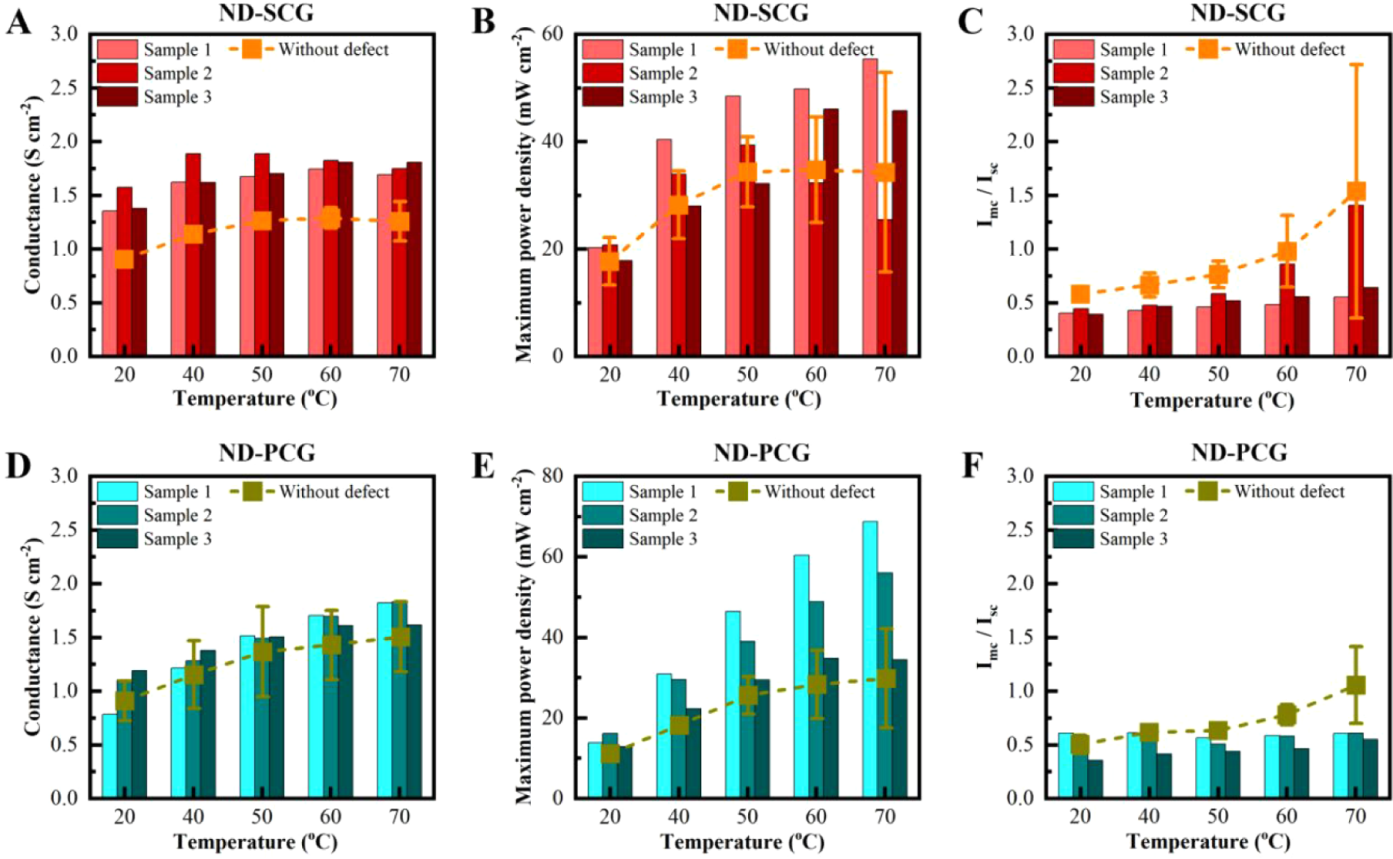
DMFC performance with defective graphene. Conductance, maximum power output, and I_MC_/I_SC_ of ND-SCGs (A–C) and ND-PCGs (D–F). The dashed lines with symbols indicate the averaged numbers with a standard deviation of SCGs and PCGs measured under the same conditions.

Remarkably, the introduced defects improved the total proton/methanol selectivity. The I_MC_/I_SC_ ratio of ND-SCGs (Figure 4C) is lower than that of SCGs. At 50 °C, the averaged I_MC_/I_SC_ ratio of ND-SCGs is 0.63 ± 0.20, which is 37% lower than that of SCGs. Across the entire temperature range, the I_MC_/I_SC_ ratio of ND-SCGs is 32–45% lower than that of SCGs. The reduced I_MC_/I_SC_ ratio of ND-SCGs indicates an enhanced proton/methanol selectivity for permeation from the anode to the cathode. With the improvement in proton conductance, power output, and selectivity, the introduction of defects has proven to be an effective method for enhancing DMFCs with graphene membranes.

ND-PCG shows a greater enhancement in maximum power output, though a smaller improvement in conductance is measured, compared to the enhancement observed for ND-SCG, attributed to a higher proton transport over methanol crossover. Figure 4D shows the conductance of ND-PCGs. At 50 °C, the average conductance of ND-PCGs is 1.50 ± 0.01 S cm^–2^, which is 10% higher than that of PCGs. For the elevated operating temperatures, the conductance improvement ranges from 10 to 16%. Although the conductance improvement in ND-PCGs is lower than in ND-SCGs, the power output of ND-PCGs more significantly increased by 48% at 50 °C (38.3 ± 8.5 mW cm^–2^) and 77% at 70 °C (53.1 ± 17.3 mW cm^–2^), as shown in Figure 4E. In contrast, with a lower increase in proton conductance, the averaged I_MC_/I_SC_ ratio of ND-PCGs is only 23% lower than that of PCGs at 50 °C and up to 56% lower at 70 °C. This indicates a greater enhancement in proton transport vs methanol crossover and therefore an overall increase of DMFC membrane stability concerning the operation temperature for ND-PCGs compared to ND-SCGs, as expected.

In summary, the introduction of defects effectively improves proton conductance and selectivity for both SCG and PCG membranes. The estimation of proton conductance enhancement in defected samples is based on the comparison with samples exhibiting intrinsic defects/cracks; thus, it is difficult to precisely estimate the intrinsic improvement attributable to lattice defects alone. The improvement of the proton conductance is more pronounced in SCGs, while the enhancement in power output and reduction in methanol crossover are more significant in PCGs, despite the similar defect density. We attribute this to the multilayer patches in PCGs, which not only enhance the stability of the graphene samples but also, we assume, shade defect formation, leading to improved proton selectivity—a desirable attribute for DMFC membrane applications. Proton conductance improvement is more pronounced in SCGs, while the enhancement in power output and reduction in methanol crossover are more significant in PCGs, despite the similar defect density. We attribute this to the multilayer patches in PCGs, which enhances the stability of the graphene samples and shades defect formation during the plasma treatment.

### DMFC under High Methanol Concentration

The increase in methanol concentration poses challenges for the membrane to prevent crossover permeation, as it leads to a higher concentration of active fuel in the water mixture (catalyst poisoning). Given the higher proton/methanol selectivity of ND-PCGs compared to ND-SCGs, it is expected that ND-PCGs would exhibit greater tolerance to higher methanol concentrations. Figure 5A presents the P–I curves of SCGs, ND-SCGs, PCGs, and ND-PCGs under 1 and 5 M methanol concentrations. In comparison to the 1 M methanol/water condition, there is a consistent decrease in maximum power output for all membranes with a 5 M methanol/water mixture, indicating a more severe methanol crossover at higher methanol concentrations. At room temperature, SCG-DMFCs exhibit higher power outputs compared to PCG-DMFCs with 5 M methanol/water mixture (Table S1). However, at higher temperatures (above 50 °C), the difference becomes smaller. This trend aligns with the results obtained under the 1 M methanol/water mixture, indicating that SCGs have a relatively higher likelihood of methanol leakage at higher temperatures. To facilitate comparison, we calculated the relative variation of the maximum power output under 5 M methanol compared to 1 M methanol (Figure 5B,C). The variation is larger for ND-SCGs compared to SCGs, except at 20 °C. Specifically, at 50 °C, the average variation for ND-SCGs is −32.1%, while for SCGs it is −16.4%. This indicates that methanol permeation becomes more severe at higher methanol concentrations, and the introduced defects in SCGs decrease the tolerance to methanol concentration. In contrast, at 50 °C, the average variation for ND-PCGs is −22.5%, while for PCGs it is −24.8%.

**Figure 5:**
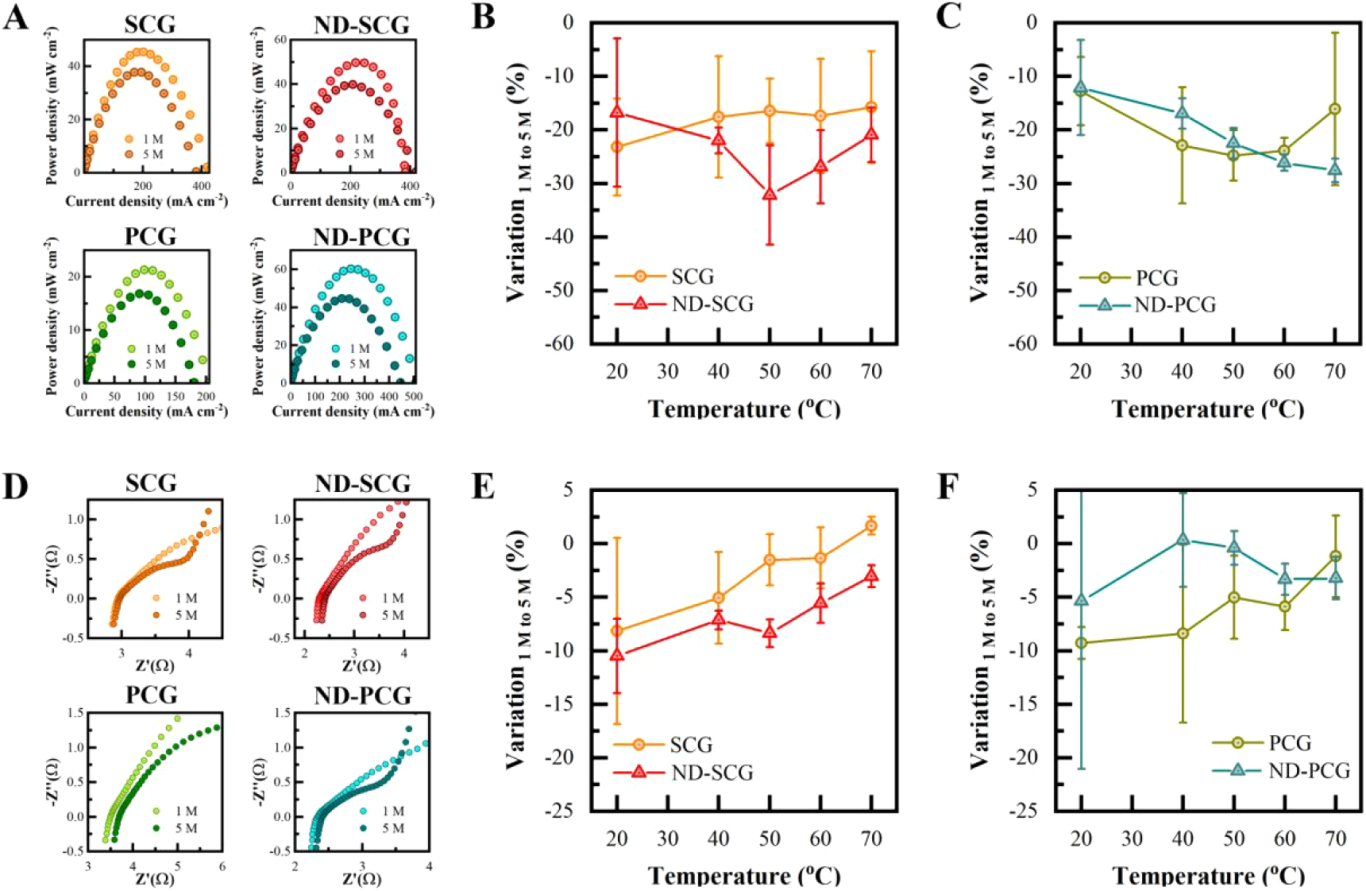
DMFC performance comparison under 5 M methanol with 1 M methanol. (A) Power–current (P–I) plots of DMFCs operated at 60 °C using SCG, ND-SCG, PCG, and ND-PCG membranes under 1 and 5 M methanol concentrations. (B) Average variation of maximum power output for 5 M methanol compared to 1 M methanol for SCGs and ND-SCGs. (C) Average variation of maximum power output for 5 M methanol compared to 1 M methanol for PCGs and ND-PCGs. (D) Nyquist plots of DMFCs operated at 60 °C under open-circuit voltage conditions using SCG, ND-SCG, PCG, and ND-PCG membranes with 1 and 5 M methanol concentrations. (E) Average variation of membrane conductance for 5 M methanol compared to 1 M methanol for SCGs and ND-SCGs. (F) Average variation of membrane conductance for 5 M methanol compared to 1 M methanol for PCGs and ND-PCGs.

Insights into the resistance of membranes and electrodes can be obtained from electrochemical impedance spectroscopy (EIS). Figure 5D presents the Nyquist plots of SCG, ND-SCG, PCG, and ND-PCG membranes with 1 and 5 M methanol under open circuit voltage conditions. The intercepts on the *X*-axis indicate the membrane resistance, while the diameter of the semicircle represents the anode resistance. For DMFCs operated with 5 M methanol, a slight increase in resistance is observed for all samples, which can be attributed to the dehydration of Nafion layers, leading to increased resistance. Figure 5E shows the relative variation in switching from 1 to 5 M methanol concentration for SCGs and ND-SCGs. The variation is larger for ND-SCGs compared to SCGs (Table S2). Specifically, at room temperature, the average variation for SCGs is approximately 8.2%, while for ND-SCGs it is around 10.5%. In contrast, the average variation for PCGs is approximately 9.3%, while for ND-PCGs it is around 5.3% (Figure 5F). The reduced variation for ND-PCGs indicates an improvement in membrane tolerance to methanol concentration, which is consistent with the improvement in power output. Moreover, the semicircles corresponding to the anode resistance in Figure 5D are smaller for the 5 M methanol concentration compared to 1 M, indicating a faster reaction rate. This increased reaction rate at the anode is expected to enhance the proton generation rate, thereby potentially improving fuel cell performance. However, the permeation of methanol leads to a decrease in performance. Figures S6–S9 display the Nyquist plots measured under 200 mV, which show both semicircles for the anode and cathode. For all samples, the second semicircle attributed to the cathode oxygen reduction reaction is larger for the 5 M methanol concentration compared to 1 M methanol, indicating higher resistance. This can be attributed to an increased methanol permeation, which competes with oxygen at the cathode.

In summary, increasing the methanol concentration from 1 to 5 M results in an increased anode reaction rate and methanol crossover permeation rate. After plasma treatment, SCGs exhibit reduced stability with an increase in methanol concentration, while PCGs show higher overall performances. This observation aligns with the previous discussion on proton/methanol selectivity. ND-PCGs demonstrate higher proton/methanol selectivity under 1 M conditions, leading to greater tolerance for methanol concentration. Attempts were made to measure DMFCs under methanol concentrations higher than 5 M (up to 10 M); however, the DMFCs did not function with an open-circuit voltage below 250 mV because of the swelling of dehydrated Nafion layers in the 10 M methanol concentration.

## Conclusion

This study examines the combined effect of graphene monocrystallinity (versus polycrystallinity) and nitrogen doping—associated with vacancy defects identified by Raman spectroscopy—on how derivatization by nitrogenation and the internal structure of graphene membranes influence the performance of direct methanol fuel cells (DMFCs), particularly considering the role of multilayer “shading” patches. Single-crystalline graphene (SCG) demonstrated higher proton conductance at temperatures lower than 50 °C compared to polycrystalline graphene (PCG). However, SCG exhibited higher methanol leakage at high temperatures, indicating lower tolerance to elevated operating temperatures, presumably through the formation of cracks in the graphene during the membrane electrode assembly. We assume that the multilayer patches on PCG, as the major difference, stabilize the membrane. Introducing defects through nitrogen plasma treatment increased the conductance of both SCG and PCG. However, ND-SCG membranes experienced higher methanol crossover, while ND-PCG membranes showed lower methanol permeation and higher power output. These findings highlight the fact that the multilayer patches contribute significantly to shade the defect formation, introducing more proton-selective pathways. The shading effect and stabilization effect by multilayer patches therefore represents important structural properties of CVD materials when integrated in fuel cells and presumably in other energy devices such as electrolyzers or batteries as an integral part promoting higher membrane robustness and proton selectivity at the same time.

## Methods

### Materials and Chemicals

Large-area, high-quality single-crystal graphene samples, specifically multilayer-free graphene, were grown epitaxially on Cu(111) foils using the chemical vapor deposition (CVD) method.^31^ Commercially available polycrystalline Cu foils, with dimensions of decimeter scale, were placed in a homemade low-pressure CVD system equipped with a 6-in. quartz tube. To promote anomalous Cu grain growth and facilitate the formation of large-area Cu(111) single crystals, an asynchronous heating process was employed, creating a temperature gradient on the Cu foils.^32^ The heating and annealing process took place under Ar gas (1000 sccm, 500 Pa), which enabled the surface cleaning of Cu foils and passivation of active sites to suppress adlayer formation and nucleation density. Subsequently, with the temperature maintained at 1020 °C, graphene growth occurred using a gas mixture of H_2_ (500 sccm) and CH_4_ (0.8 sccm). For the polycrystalline graphene with multilayers, CVD graphene on Cu film was purchased from Graphenea. The methanol/water mixtures were prepared using ultrapure water from the Millipore Milli-Q Gradient A10 system (18.2 MΩ cm^–1^) and methanol purchased from VWR Chemicals (∼100%). Ammonium persulfate (APS) obtained from Sigma-Aldrich was used to prepare a 0.5 M APS solution with ultrapure water. A porous polycarbonate membrane (PC, Whatman Nuclepore track-etch membrane), with 2 μm holes was purchased from Cytiva. Additional materials for the fabrication of MEAs, including Nafion solution (D521), Nafion 117 membrane, catalyst electrodes, and PTFE gasket were purchased from Fuel Cell Store. Soft replica rubber was purchased from Flexbar Machine Corp, and the electrolyzer was purchased from Dioxide Materials (with a stainless steel cathode and titanium anode). Poly­(methyl methacrylate) (PMMA) was purchased from Allresist GmbH (6% in anisole, AR-P 662.06), and epoxy resin with curing agent was purchased from GENTEC, was also utilized in the experimental setup.

### Plasma Treatment

The treatment involving a capacitively coupled plasma system was conducted using a Diener electronic apparatus with a radio frequency (RF) of 40 kHz and a maximum power of 200 W. The chamber used in the process has a detection limit of 0.2 mbar. Prior to the treatment, the chamber was initially maintained at a high vacuum state (<0.2 mbar) for 5 min to eliminate any unwanted gases. Subsequently, the chamber was flushed with N_2_ at 0.7 mbar for 1 min, followed by vacuuming to reduce the pressure to 0.2 mbar for a total of six cycles. This procedure ensured that only the desired atmosphere was present in the chamber. For the nitrogen plasma treatment, specific conditions were applied, including a pressure of 0.7 mbar, power of 16 W, and an exposure time of 45 s.

### Graphene Composite Membrane Electrode Assembly (MEA) Fabrication

To prepare the Nafion/graphene/Cu composite membrane, a 1.2 × 1.2 cm square of CVD graphene on Cu is initially spin-coated with Nafion solution at 2000 rpm for 1 min. The sample is then baked on a hot plate at 80 °C for 30 min. Next, the Nafion/graphene/Cu layer is placed onto a porous polycarbonate membrane with pore diameters of 2 μm and a porosity of ∼13%, serving as a support. The Cu layer is completely etched by immersing the Nafion/graphene/Cu/PC sample in a 5 M APS solution. Afterward, the Nafion/graphene/PC composite membrane is rinsed and stored in 0.1 M HCl.

To assemble the membrane for measurements in DMFCs, the Nafion/graphene/PC membrane is positioned between two PTFE gasket sheets with the assistance of soft replica rubber, ensuring that no rubber is present on the top of the graphene layer. The replica rubber is cured at ambient temperature for approximately 15 min. In order to strengthen the edge of the Nafion/graphene membrane, the circular edge is sealed with PTFE by applying replica rubber paste. It is important to note that the casted rubber should have a thin thickness to ensure proper contact between the electrode and metal plates.

To remove the gas trapped in the PC membrane, 30 μL of Nafion solution is cast on the backside of the PC support, followed by baking at 80 °C for 30 min, repeated twice. Prior to the measurements in DMFCs, gas diffusion electrodes with catalysts loaded are installed on both sides of the assembly by hot pressing at 80 °C for 1 h. The anode is loaded with Pt/Ru electrodes at a rate of 4 mg/cm^–2^, while the cathode is loaded with Pt at the same rate.

The aforementioned steps ensure the successful preparation and assembly of the Nafion/graphene composite membrane for use in DMFC measurements, providing the necessary structure and electrode configuration for the fuel cell system.

### Raman Spectroscopy and Scanning Electron Microscopy

Raman spectroscopy measurements were conducted using the alpha300 R-Confocal Raman Imaging system manufactured by WITec. The system uses a laser with a wavelength of 457 nm. Prior to the Raman measurements, the CVD graphene was transferred onto a SiO_2_/Si wafer using the PMMA-assisted method. The SiO_2_ layer had a thickness of 285 nm. All Raman tests were performed under ambient conditions.

To obtain the scanning electron microscope (SEM) image, an FEI NOVA NanoSEM 200 instrument was used. This microscope allowed for high-resolution imaging of the sample morphology and structure.

### Fuel Cell Operation and Measurements

Electrochemical measurements were carried out using a potentiostat (PGSTAT204) equipped with an electrochemical impedance spectroscopy module (FRA32M) and a 10 A current booster. A titanium anode plate and a cathode made from 904L stainless steel were utilized in the electrolyzer for DMFC measurements. The cells were operated using a methanol/water mixture flowing through the anode at a rate of 30 mL min^–1^.

To ensure measurement reliability, all cells underwent a series of initial steps. These steps included five rounds of cyclic voltammetry (CV) scanning from 0 to 1.5 V at a scanning speed of 10 mV/s, cathode starvation with N_2_ gas instead of O_2_, and a 20 min run at 200 mV. The open circuit voltage was determined by fixing the cell current to 0 and recording the voltage after 5 min of no voltage variation.

Polarization curves were obtained once the cells were fully stabilized, with the current variation being less than 10 μA min^–1^. The curves were generated by linearly sweeping from the open circuit voltage to the short circuit voltage at a scan rate of 10 mV/s. Resistance values were determined by reading the intercept from the Nyquist plot of the electrochemical impedance spectrum. The impedance spectrum was measured with frequencies ranging from 1 kHz to 0.1 Hz and an amplitude of 10 mV under open circuit conditions.

The methanol crossover current was measured using linear sweep voltammetry from 0 to 1.2 V against both the cathode and anode. The scan rate was set at 5 mV/s, and the methanol flow rate was maintained at the anode, while nitrogen flow was maintained at the cathode. The limiting current observed during the measurement was taken as an indicator of the methanol crossover rate.

## Supplementary Material



## Notes

Schematic illustration of the fabrication process; optical image of porous polycarbonate; construction of DMFC; characterizations of graphene membranes; the averaged maximum power density of SCGs, D-SCGs, PCGs, and D-PCGs; the averaged conductance of SCGs, D-SCGs, PCGs, and D-PCGs; Nyquist plots (PDF)

## References

[1] Mogg L. (2019). Perfect proton selectivity in ion transport through two-dimensional
crystals. Nat. Commun..

[2] Hu S. (2014). Proton transport through one-atom-thick crystals. Nature.

[3] Kidambi P. R., Chaturvedi P., Moehring N. K. (2021). Subatomic species transport through
atomically thin membranes: Present and future applications. Science.

[4] Arico A. S., Srinivasan S., Antonucci V. (2001). DMFCs: From fundamental aspects to
technology development. Fuel Cells.

[5] Steele B. C. H., Heinzel A. (2001). Materials for fuel-cell
technologies. Nature.

[6] Park H. B., Kamcev J., Robeson L. M., Elimelech M., Freeman B. D. (2017). Maximizing the right stuff: The trade-off
between membrane
permeability and selectivity. Science.

[7] Ng W. W., Thiam H. S., Pang Y. L., Chong K. C., Lai S. O. (2022). A state-of-art
on the development of Nafion-based membrane for performance improvement
in direct methanol fuel cells. Membranes.

[8] Allioux F. M. (2017). Insights into free volume
variations across ion-exchange membranes
upon mixed solvents uptake by small and ultrasmall angle neutron scattering. ACS Appl. Mater. Interfaces.

[9] Sazali N., Salleh W. N. W., Jamaludin A. S., Razali M. N. M. (2020). New perspectives
on fuel cell Technology: A brief review. Membranes.

[10] Harun N. A. M., Shaari N., Zaiman N. F. H. N. (2021). A
review of alternative polymer electrolyte
membrane for fuel cell application based on sulfonated poly­(ether
ether ketone). Int. J. Energy Res..

[11] Jiang Z. Q., Zhao X. S., Fu Y. Z., Manthiram A. (2012). Composite
membranes based on sulfonated poly­(ether ether ketone) and SDBS-adsorbed
graphene oxide for direct methanol fuel cells. J. Mater. Chem..

[12] Duan Y., Ru C., Li J., Sun Y.-N., Pu X., Liu B., Pang B., Zhao C. (2022). Enhancing proton conductivity
and methanol resistance of SPAEK membrane by incorporating MOF with
flexible alkyl sulfonic acid for DMFC. J. Membr.
Sci..

[13] Bukola S., Liang Y., Korzeniewski C., Harris J., Creager S. (2018). Selective
proton/deuteron transport through nafion|graphene|nafion sandwich
structures at high current density. J. Am. Chem.
Soc..

[14] Achtyl J. L. (2015). Aqueous proton transfer across single-layer graphene. Nat. Commun..

[15] Walker M. I., Weatherup R. S., Bell N. A. W., Hofmann S., Keyser U. F. (2015). Free-standing
graphene membranes on glass nanopores for ionic current measurements. Appl. Phys. Lett..

[16] Chaturvedi P. (2019). Ionic
conductance through graphene: Assessing its applicability as
a proton selective membrane. ACS Nano.

[17] Bukola S., Beard K., Korzeniewski C., Harris J. M., Creager S. E. (2019). Single-layer
graphene sandwiched between proton-exchange membranes for selective
proton transmission. ACS Appl. Nano Mater..

[18] Sirkin Y. A. P., Tagliazucchi M., Szleifer I. (2020). Transport in nanopores and nanochannels:
some fundamental challenges and nature-inspired solutions. Mater. Today Adv..

[19] Griffin E. (2020). Proton and Li-ion permeation
through graphene with eight-atom-ring
defects. ACS Nano.

[20] Wang L. D. (2017). Fundamental transport
mechanisms, fabrication and potential applications
of nanoporous atomically thin membranes. Nat.
Nanotechnol..

[21] Jang D., Idrobo J. C., Laoui T., Karnik R. (2017). Water and solute transport
governed by tunable pore size distributions in nanoporous graphene
membranes. ACS Nano.

[22] Sun P. Z., Xiong Q., Bera A., Timokhin I., Wu Z. F., Mishchenko A., Sellers M. C., Liu B. L., Cheng H. M., Janzen E., Edgar J. H. (2023). Unexpected catalytic
activity of nanorippled graphene. Proc. Natl.
Acad. Sci. U. S. A..

[23] Holmes S. M., Balakrishnan P., Kalangi V. S., Zhang X., Lozada-Hidalgo M., Ajayan P. M., Nair R. R. (2017). 2D crystals significantly
enhance the performance of a working fuel cell. Adv. Energy Mater..

[24] Yan X. H., Wu R. Z., Xu J. B., Luo Z. T., Zhao T. S. (2016). A monolayer
graphene - Nafion sandwich membrane for direct methanol fuel cells. J. Power Sources.

[25] Sun L. Z. (2022). Toward epitaxial growth
of misorientation-free graphene on Cu(111)
foils. ACS Nano.

[26] Eckmann A. (2012). Probing the nature of
defects in graphene by Raman spectroscopy. Nano
Lett..

[27] Tuinstra F., Koenig J. L. (1970). Raman Spectrum Of
Graphite. J.
Chem. Phys..

[28] Ferrari A.
C., Meyer J. C., Scardaci V., Casiraghi C., Lazzeri M., Mauri F., Piscanec S., Jiang D., Novoselov K. S., Roth S. (2006). Raman spectrum of graphene
and graphene layers. Phys. Rev. Lett..

[29] Metzger N., Vlassiouk I., Smirnov S., Mariscal G., Spragg R., Li X. (2023). Experimental studies of graphene-coated Ppolymer electrolyte
membranes for direct methanol fuel cells. J.
Electrochem. Energy Convers. Storage.

[30] Zhang W. (2024). Role of vacancy defects and nitrogen dopants for the reduction of
oxygen on graphene. ACS Catal..

[31] Sun L., Chen B., Wang W., Li Y., Zeng X., Liu H., Liang Y., Zhao Z., Cai A., Zhang R. (2022). Toward epitaxial growth of misorientation-free
graphene on Cu(111)
foils. ACS Nano.

[32] Li Y., Sun L., Chang Z., Liu H., Wang Y., Liang Y., Chen B., Ding Q., Zhao Z., Wang R. (2020). Large single-crystal Cu foils with high-index facets by strain-engineered
anomalous grain growth. Adv. Mater..

