# Supplementary material for: Vacancy Defects and Multilayer Shading in Graphene Monolayers: Enhancing Proton Transport in Centimeter-Sized Direct Methanol Fuel Cells
Source: ACS Appl Mater Interfaces. 2025 Nov 5;17(46):63438–47. doi: 10.1021/acsami.5c16491 (PMC12635971; doi:10.1021/acsami.5c16491)
Supplement: Supplementary file 1 [file am5c16491_si_001.pdf]

Supporting information

for

Vacancy defect and multilayer shading in graphene monolayers: enhancing proton transport in centimeter-sized direct methanol fuel cells

Weizhe Zhang[1], Xiaoting Liu[2,3], Buhang Chen[2,3], Luzhao Sun[2,3], Zhongfan Liu[2,3], Grégory F. Schneider[1]*

[1] Leiden Institute of Chemistry, Leiden University, Faculty of Science, Einsteinweg 55, 2333CC Leiden, The Netherlands

[2] Center for Nanochemistry, Beijing Science and Engineering Center for Nanocarbons, Beijing National Laboratory for Molecular Sciences, College of Chemistry and Molecular Engineering, Academy for Advanced Interdisciplinary Studies, Peking University, Beijing, China

[3] Beijing Graphene Institute (BGI), Beijing, China

* to whom correspondence should be addressed: g.f.schneider@chem.leidenuniv.nl

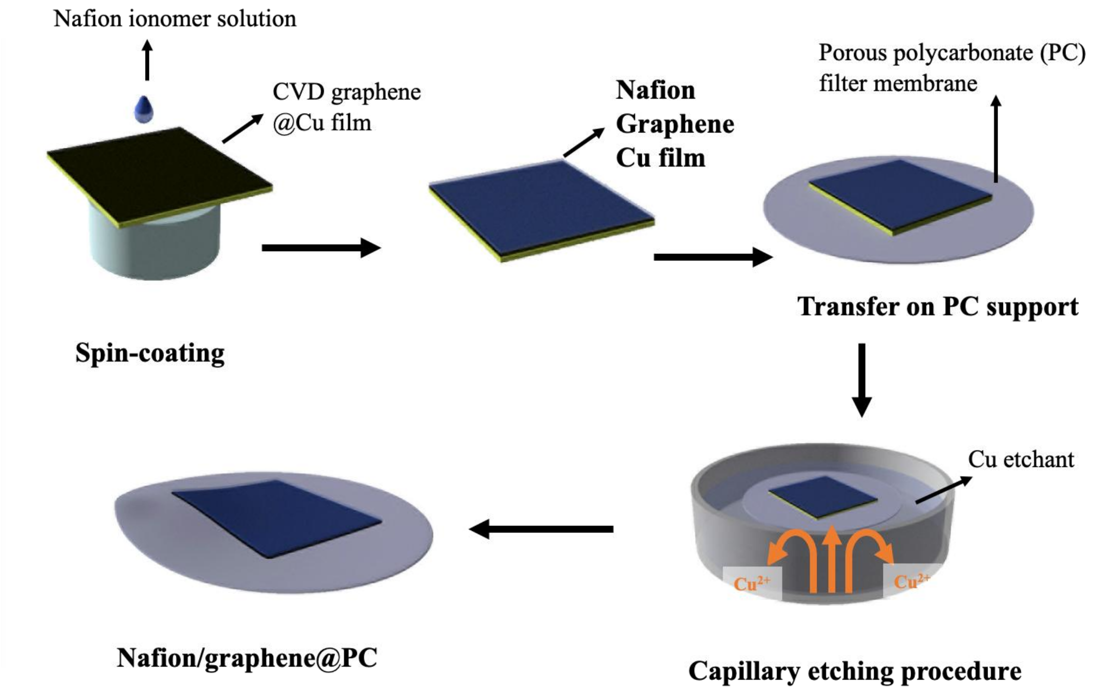

Figure S1. Schematic illustration of the fabrication process for the Nafion/graphene/polycarbonate (PC) composite membrane. A 1.2 × 1.2 cm^2 sheet of CVD-grown graphene on copper (Cu) is first spin-coated with Nafion solution at 2000 rpm for 1 minute. The sample is then baked on a hot plate at 80 °C for 30 minutes. Subsequently, the Nafion/graphene/Cu film is transferred onto a porous polycarbonate membrane (pore diameter: 2 μm), which acts as a mechanical support. The underlying Cu is fully etched by floating the structure at the air/water meniscus with water containing 0.5 M ammonium persulfate (APS) solution. Finally, the resulting Nafion/graphene/PC composite membrane is rinsed and stored in 0.1 M HCl.

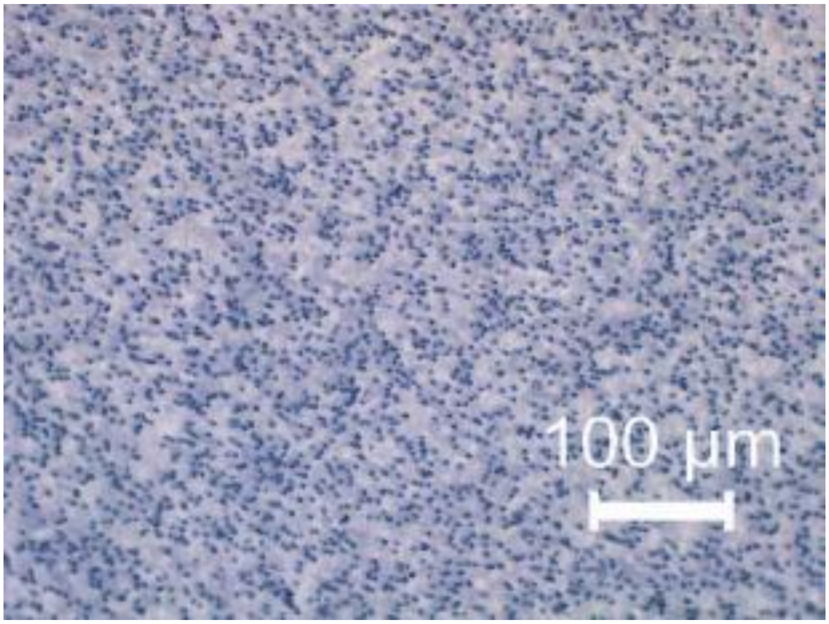

Figure S2. Optical image of porous polycarbonate (PC) membrane. The black spots (~2 μm in diameter) are the straight pores in the PC membrane (the thickness of the membrane is ~10 μm) and the areal ratio of pores to the total membrane area is ~13%.

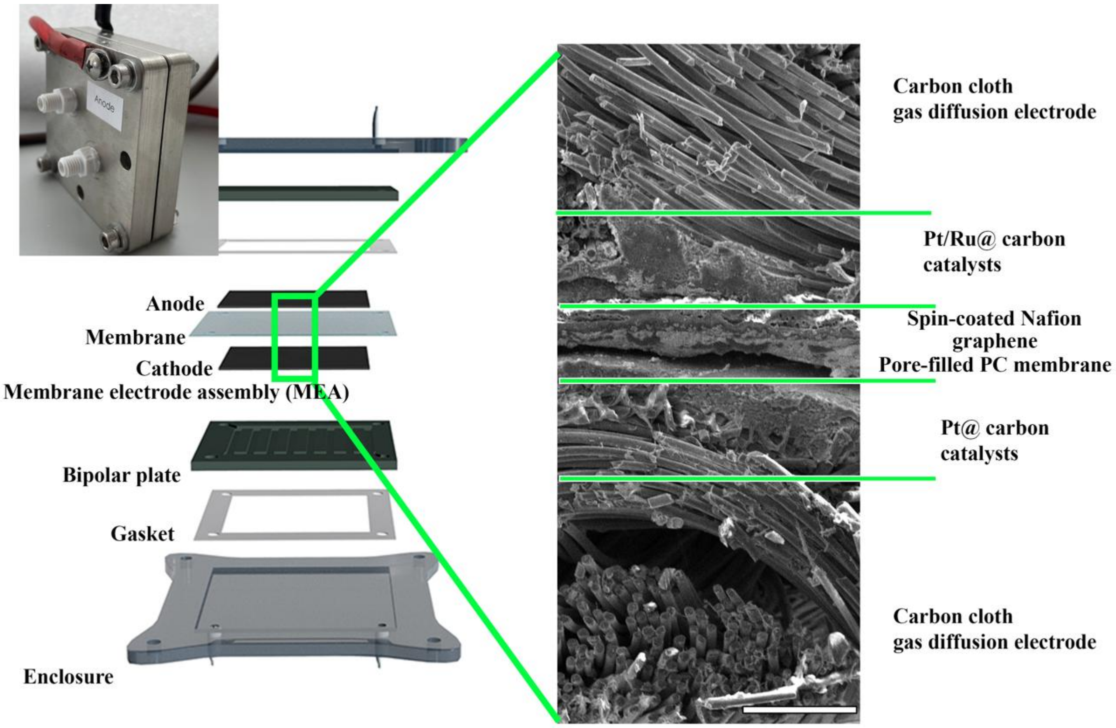

Figure S3. Construction of the DMFC. The DMFC consists of enclosed bipolar plates

for electron collection, a PTFE gasket, and a membrane electrode assembly (MEA) where electrochemical reactions occur. The right panel shows an SEM image of an MEA cross-section, with the graphene-based MEA highlighted by the green line. Scale bar: 100 μm.

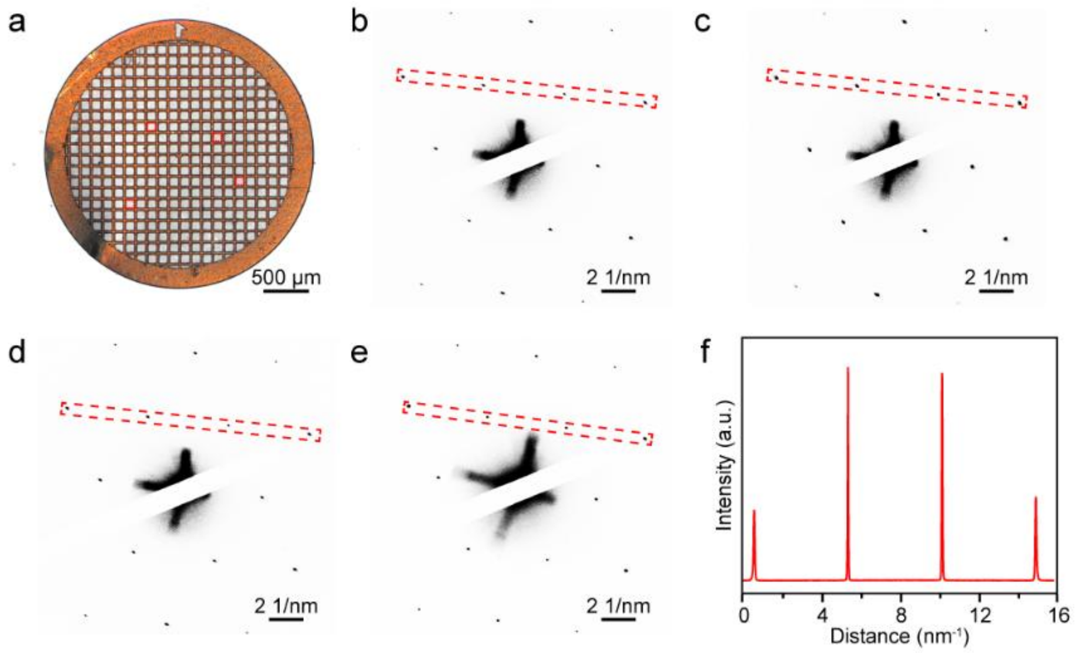

Figure S4. Characterization of graphene lattice orientation. (a) Optical microscopy image of the suspended graphene transferred onto a TEM grid. (b-e) Typical selected area electron diffraction (SAED) patterns of the suspended single-crystal graphene measured at the marked position in (a). (f) The intensity profile of the diffraction pattern along the red dashed line in (e). This figure was adapted from our previous work[1].

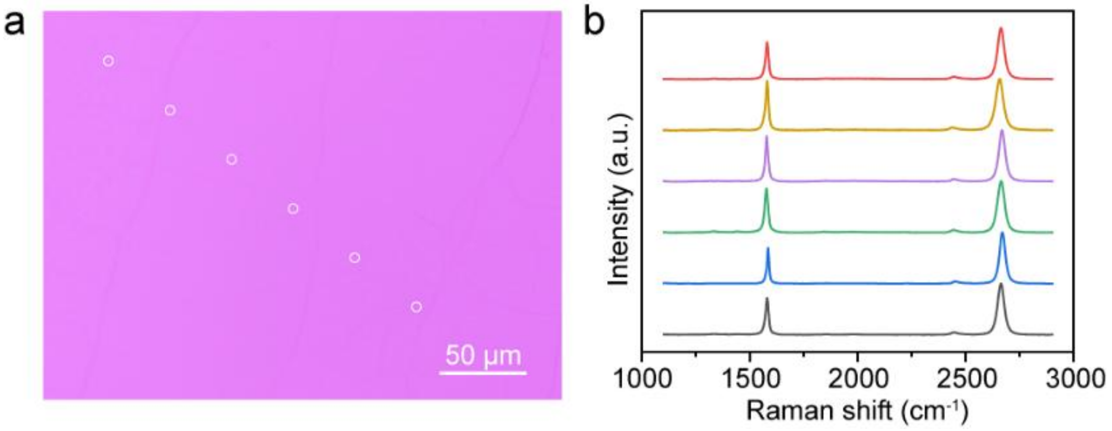

Figure S5 Characterization of the high-quality graphene film. (a) Optical microscopy image of monolayer graphene transferred onto a SiO_2/Si substrate. (b) Representative Raman spectra of the as-transferred graphene measured at the marked position in (a). This figure was adapted from our previous work[1].

The DMFC power output and membrane conductance were measured for membranes fabricated from single-crystalline graphene (SCG), polycrystalline graphene (PCG), nitrogen-doped single-crystalline graphene (ND-SCG), and nitrogen-doped polycrystalline graphene (ND-PCG) using three independent samples for each type. The steady-state results, measured as a function of temperature, are summarized in Tables S1 and S2, with values reported as mean ± standard deviation.

Table S1. Averaged maximum power density of SCGs, D-SCGs, PCGs, and D-PCGs.

	The averaged maximum power density (mW cm^{-2})									
	20 °C		40 °C		50 °C		60 °C		70 °C	
	1 M	5 M	1 M	5 M	1 M	5 M	1 M	5 M	1 M	5 M
SCG	18.20 ± 4.4	13.9 ± 3.4	28.7 ± 6.3	23.4 ± 4.3	34.9 ± 6.5	29.4 ± 7.3	35.3 ± 9.8	28.9 ± 7.8	34.8 ± 18.5	28.8 ± 15.2
D-SCG	19.6	16.4	34.1	26.6	40.0	27.1	42.7	31.3	42.2	32.9

	± 1.6	± 3.3	± 6.2	± 4.6	± 8.2	± 6.5	± 9.2	± 8.0	± 15.3	± 10.3
PCG	11.3	9.9	18.4	14.2	25.8	19.5	28.5	21.6	30.0	24.5
	± 0.7	± 1.3	± 1.3	± 2.8	± 4.7	± 4.1	± 8.4	± 6.0	± 12.3	± 7.7
D-PCG	14.3	12.6	27.6	23.0	38.3	29.8	48.0	35.6	53.1	38.3
	± 1.7	± 1.8	± 4.6	±4.6	± 8.5	± 7.4	± 12.8	± 9.9	± 17.3	± 11.6

Table S2. The averaged conductance of SCGs, D-SCGs, PCGs and D-PCGs.

	The averaged conductance (S cm^{-2})									
	20 °C		40 °C		50 °C		60 °C		70 °C	
	1 M	5 M	1 M	5 M	1 M	5 M	1 M	5 M	1 M	5 M
SCG	0.93	0.85	1.16	1.10	1.29	1.27	1.32	1.30	1.28	1.30
	± 0.05	± 0.11	± 0.01	± 0.05	± 0.03	± 0.04	± 0.10	± 0.06	± 0.18	± 0.18
D-SCG	1.44	1.20	1.71	1.59	1.76	1.61	1.79	1.69	1.75	1.70
	± 0.12	± 0.07	± 0.15	± 0.13	± 0.11	± 0.08	± 0.04	± 0.04	± 0.06	± 0.04
PCG	0.92	0.83	1.16	1.05	1.37	1.30	1.44	1.35	1.51	1.50
	± 0.19	± 0.16	± 0.32	± 0.22	± 0.42	± 0.40	± 0.32	± 0.28	± 0.33	± 0.38
D-PCG	1.03	0.95	1.29	1.29	1.50	1.50	1.67	1.62	1.76	1.70
	± 0.22	± 0.12	± 0.08	± 0.03	± 0.01	± 0.03	± 0.05	± 0.07	± 0.12	± 0.12

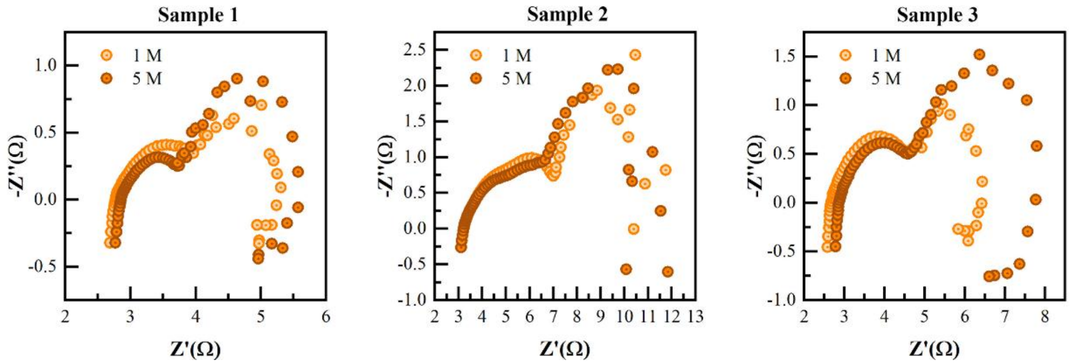

Figure S6. Nyquist plots of SCGs under 1 M and 5 M methanol, at 60 °C.

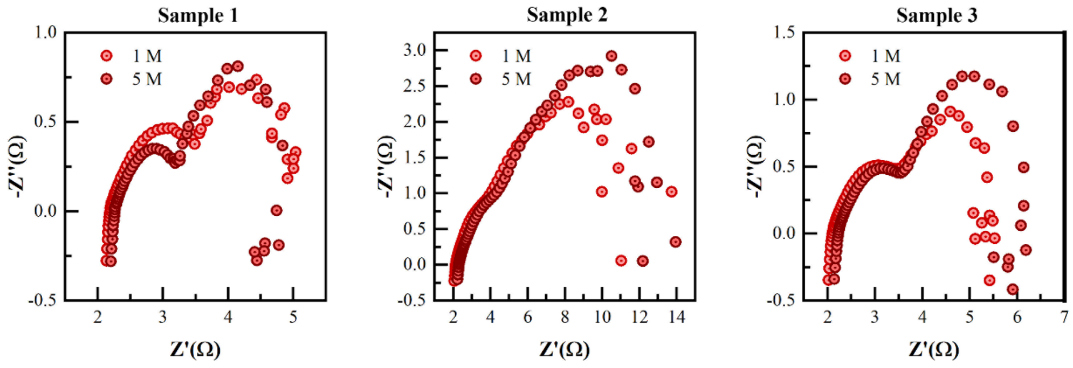

Figure S7. Nyquist plots of D-SCGs under 1 M and 5 M methanol, 60 °C.

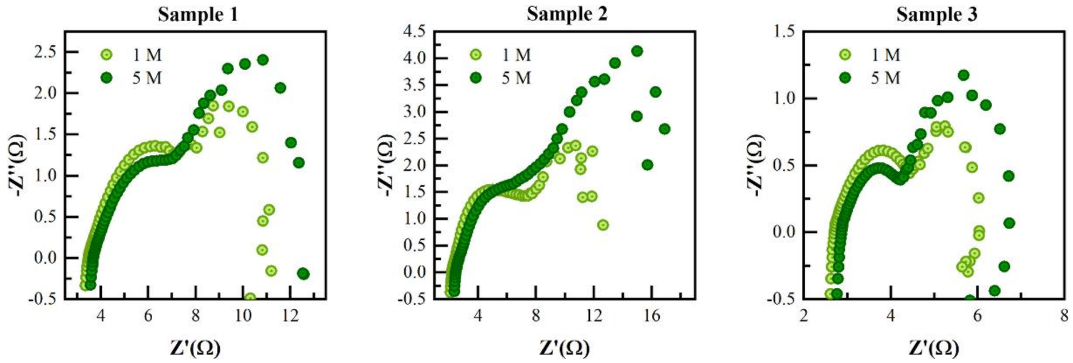

Figure S8. Nyquist plots of PCGs under 1 M and 5 M methanol, at 60 °C.

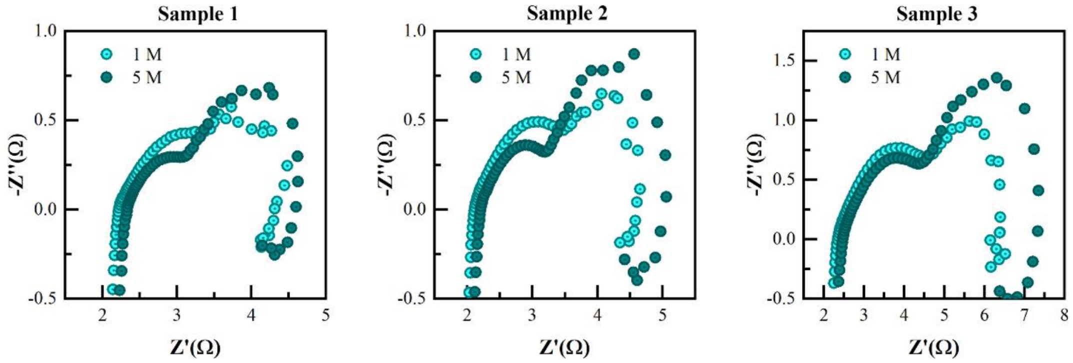

Figure S9. Nyquist plots of D-PCGs under 1 M and 5 M methanol, at 60 °C.

Reference.

(1) Zhang, W.; Makurat, M.; Liu, X.; Kang, X.; Liu, X.; Li, Y.; Kock, T. J. F.; Leist, C.; Maheu, C.; Sezen, H.; et al. Giant proton transmembrane transport through sulfophenylated graphene in a direct methanol fuel

cell. 2023; preprint: arXiv:2308.16112.
